# *Escherichia coli* O78 isolated from septicemic lambs shows high pathogenicity in a zebrafish model

**DOI:** 10.1186/s13567-016-0407-0

**Published:** 2017-01-25

**Authors:** Cecilie K. Kjelstrup, Amelia E. Barber, J. Paul Norton, Matthew A. Mulvey, Trine M. L’Abée-Lund

**Affiliations:** 1 Department of Food Safety and Infection Biology, Norwegian University of Life Sciences, P.O. Box 8146 Dep, 0033 Oslo, Norway; 2 Division of Microbiology and Immunology, Pathology Department, University of Utah, Salt Lake City, UT USA

## Abstract

The pathogenicity of *Escherichia coli* O78 strain K46, originally isolated from an outbreak of septicemia in neonatal lambs, was investigated in zebrafish embryo and murine models of infection. Its biofilm potential, cellulose production, and the expression of type 1 pili and curli fimbriae were measured by in vitro assays. The strain was highly pathogenic in the zebrafish embryo model of infection, where it killed all embryos within 24 h post inoculation (hpi) at doses as low as 1000 colony forming units. Zebrafish embryos inoculated with similar doses of commensal *E. coli* strains showed no signs of disease, and cleared the bacteria within 24 h. *E. coli* K46 colonized the murine gut at the same level as the uropathogenic *E. coli* (UPEC) reference strain CFT073 in CBA/J mice after oral inoculation, but infected the murine bladder significantly less than CFT073 after transurethral inoculation. Type 1 pili were clearly expressed by *E. coli* K46, while curli fimbriae and cellulose production were weakly expressed. The ability to produce biofilm varied in different growth media, but overall *E. coli* K46 was a poorer biofilm producer compared to the reference strain *E. coli* UTI89. In conclusion, the zebrafish lethality model provides further evidence that *E. coli* K46 is highly pathogenic and might be useful in future studies to identify bacterial virulence factors.

## Introduction

Though often viewed as inert commensals, some strains of *Escherichia coli* are able to cause disease. Pathogenic *E. coli* are genetically diverse and cause a variety of infections in animals and human beings. Pathogenic strains are frequently categorized into diarrheagenic *E. coli* (DEC) or extraintestinal pathogenic *E. coli* (ExPEC). DEC and ExPEC are further subcategorized into specific pathogroups based on the site or mode of infection [1]. Among the diseases caused by ExPEC are septicemia, neonatal meningitis, and urinary tract infections (UTI). Despite causing disease outside the gut, such bacteria have been shown to colonize the gastrointestinal (GI) tract of the host, in some cases even more efficiently than commensal *E. coli* strains [1]. The GI tract is the main reservoir for pathogenic *E. coli* [2], and in septicemic disease, the GI tract together with the urogenital tract, are important portals of *E. coli* entry [3].

ExPEC are genetically diverse and there are no conserved virulence factors (VFs) or genetic characteristic that defines the ExPEC pathogroup or are essential for development of disease. Instead, septicemic bacteria possess a combination of VFs within the main groups of virulence traits (i.e. the adhesins, capsular synthesis, toxins, invasins and iron uptake systems). The set of VFs present in each septicemic strain enables the bacteria to defeat the host’s defense mechanisms and cause an infection [4]. The lack of common VFs among septicemic strains and the numerous possible combinations of VFs make the recognition of septicemic strains and the assessment of their pathogenic potential challenging.

Genomic analysis can provide clues to a strain’s virulence and demonstrate its similarity to other pathogenic isolates. However, to truly assess a strain’s pathogenic potential, it must be tested in a biological assay or animal model. Cell culture as well as invertebrate and vertebrate models have been developed to study septicemic disease and ExPEC virulence specifically. These models range from murine models aimed to differentiate pathogenic from nonpathogenic *E. coli* strains [5, 6] to the unicellular amoeba model, *Dictyostelium discoideum*, measuring the level of bacterial pathogenicity [7].

Zebrafish (*Danio rerio*) embryos has been shown to be a useful model organism for the study of host-pathogen interactions and the virulence potential of ExPEC strains [8]. During the early stages of development, the zebrafish immune system is composed solely of innate defenses, including phagocytes, NK cells, complement and toll-like receptors, and this corresponds to the immune system of neonatal lambs [9–11].

*Escherichia coli* O78 strain K46 was isolated from an outbreak of septicemia in neonatal lambs in Norway in 2008 [12]. Previous genetic characterization could not explain the observed high virulence of this strain [12]. The aim of this study was to investigate the virulence potential of *E. coli* K46 in in vivo virulence assays such as a zebrafish septicemia model, a murine gut colonization model, and a murine urinary tract model, and in in vitro assays for the production of pili/fimbriae. In addition, the survival potential in the environment was examined in a biofilm assay.

## Materials and methods

### Bacterial strains

The septicemic *E. coli* K46 was used in all the experiments in this study. Two uropathogenic *E. coli* strains, CFT073 and UTI89, and four enteric *E. coli* strains, designated Ctr 1- Ctr 4, were used as reference and control strains, respectively, for one or more of the experiments in this study (Table 1).

**Table 1:** **Overview over** ***E. coli*** **strains used in the study**

Strain	Source	Characteristics	Reference
*E. coli* wildtype strains			
K46	Septicemia, lamb	O78 Tetracycline and sulfonamide resistant Phylogenetic group C^a^	[12]
Ctr 1	Feces, healthy lamb	Phylogenetic group B2	[12]
Ctr 2	Feces, healthy lamb	Phylogenetic group B1	[12]
Ctr 3	Feces, healthy lamb	Phylogenetic group B2	[12]
Ctr 4	Feces, healthy lamb	Phylogenetic group B2	[12]
CFT073	Pyelonephritis, human	O6:K2:H1 Cytotoxic	[31], [2]
UTI89	Cystitis-derived, human	O18:K1:H7	[32]
*E. coli* recombinant strains			
CFT073-Clm^R^	CFT073	Chloramphenicol resistant	[33]
K46 pGEN-GFP	K46	Expresses destabilized green fluorescent protein (GFP)	This study
Ctr 1 pGEN-GFP	Ctr 1	Expresses destabilized GFP	This study
Ctr 2 pGEN-GFP	Ctr 2	Expresses destabilized GFP	This study

^a^Previously referred to as belonging to phylogenetic group A, but is now regrouped to Group C [12].

### Media and conditions

The minimal medium M9 (6 g/L Na_2_HPO_4_, 3 g/L KH_2_PO_4_, 1 g/L NH_4_Cl, 0.5 g/L NaCl, 1 mM MgSO_4_, 0.1 mM CaCl_2_, 0.1% glucose, 0.0025% nicotinic acid and 16.5 µg/mL thiamine), YESCA (0.5 g/L yeast extract, 10 g/L casamino acids) and Luria–Bertani (LB) broth were used in one or more of the experiments. The cultures were grown overnight in an aerobic atmosphere at 37 °C shaking at 225 rpm if not otherwise specified.

In the zebrafish trial, inocula were prepared by centrifugation of 1 mL of static, 24 h M9 culture at approximately 7000 RCF for 2 min, and the pellet was washed once with 1 mL sterile PBS (Hyclone) and resuspended in 275–550 µL PBS to obtain appropriate bacterial densities for microinjection.

In the murine gut colonization trial, 12 mL of a static overnight M9 culture was centrifuged at approximately 8000 RCF for 10 min. The pellet was washed with 1 mL PBS, the centrifugation step repeated, and the pellet was resuspended in 500 µL sterile PBS. The number of colony forming units (CFU)/mL was measured after serial dilutions and plating to LB plates. For recovery of *E. coli* K46 and the reference strain CFT073 in the murine gut colonization study, tetracycline (5 mg/mL) and chloramphenicol (10 mg/mL), respectively, were added to the LB plates as selective agents.

In the mouse UTI trial, 20 mL of a static overnight M9 culture was centrifuged at approximately 8000 RCF for 10 min at 20 °C and the pellet was resuspended in 8 mL sterile PBS and diluted 1:10. Serial dilutions and plating on LB plates were done to quantify the infection dose.

### Zebrafish septicemia trials

To measure the virulence potential of *E. coli* K46 and the control strains Ctr 1 and Ctr 2, a zebrafish septicemia model was used as in [8]. Briefly, 48–54 h old zebrafish embryos were allocated in three groups of 18–20 embryos each. Each fish embryo was kept separately during the experiment. The three groups were inoculated with approximately 10^3^ CFU of *E. coli* K46, Ctr 1 or Ctr 2, into the bloodstream via the circulatory valley, resulting in rapid, systemic dispersal. Live and dead fish were counted at 6, 12, 14, 18, 24, 48 and 72 h post inoculum (hpi), but the 12, 14 and 18 time points were only included in one trial. Death was defined as the complete absence of heart rhythm and blood flow. To quantify bacterial burden during the course of infection, embryos from each infection group were collected at 6 and 12 hpi and individually homogenized with a mechanical PRO 250 homogenizer (PRO Scientific) in 0.5 mL PBS containing 0.5% Triton X-100. Homogenates were serially diluted and plated on LB agar, incubated overnight at 37 °C and CFUs were counted. Both the survival trial and the quantification trial were run in biological duplicates.

To visually track the course of infection in real time, zebrafish embryos were also injected with 1–2 × 10^3^ CFU of *E. coli* K46, Ctr 1 or Ctr 2 carrying pGEN-GFP (LVA) [8] for constitutive expression of destabilized green fluorescent protein (GFP) protein (GFP-marked strains). At 12 hpi embryos were fixed by incubation overnight at 4 °C in PBS containing 4% paraformaldehyde in a light proof container. Fixed embryos were washed in PBS containing 0.8% Triton X-100 in the dark for 4 × 20 min and then incubated for 10 min each in 30 and 50% glycerol before being transferred to a lightproof container and stored at 4 °C. Samples were imaged using an Olympus SZX10 stereomicroscope equipped with an Olympus DP72 camera.

### Murine gut colonization trials

To measure *E. coli* K46’s ability to colonize the gastrointestinal tract, two groups of five 6–7 week old female CBA/J mice (Jackson Laboratory, USA) were anesthetized by inhalation of isoflurane gas and gavaged with 50 µL of approximately 3 × 10^9^ CFU of either *E. coli* K46 (resistant to tetracycline) or CFT073-Clm^R^ (resistant to chloramphenicol). The two groups were kept in separate cages, and each mouse was marked individually. Fecal matter from each mouse was collected on the day of inoculation to ensure the absence of any tetracycline and chloramphenicol resistant bacteria in the commensal microbiota. Fecal samples were collected daily for 10 consecutive days and after 12 days post inoculum (dpi). Approximately 100 mg of feces/mouse/day was resuspended in 900 µL 0.7% NaCl. Serial dilutions were plated on LB agar with either chloramphenicol or tetracycline and incubated overnight at 37 °C for quantification of CFT073-Clm^R^ and *E. coli* K46, respectively. At 14 dpi the mice were anesthetized and euthanized by cervical dislocation. The liver, kidney, bladder and intestines from the mice with *E. coli* K46 were homogenized in 0.25% Triton X-100, serially diluted in 0.7% NaCl and plated on LB plates with tetracycline. The mice showed no overt signs of sickness throughout the trial.

### Mouse UTI trials

Transurethral catheterization as described by Wiles et al. [13] were performed in order to investigate whether *E. coli* K46 could be an original urinary tract pathogen (i.e. that the urogenital tract was the port of entry in the diseased lambs). Eleven 7–8 week old female CBA/J mice (Jackson Laboratory, USA) were anesthetized by isoflurane inhalation and slowly inoculated through the urethra with a 50 µL bacterial suspension of approximately 10^5^–10^6^ CFU of *E. coli* K46. The mice were euthanized 3 days later by cervical dislocation under anesthesia, and the bladder and left kidney were homogenized separately in 1 mL of 0.025% Triton X-100. The suspensions were serially diluted, spread onto LB agar plates, and incubated for about 18 h at 37 °C, and CFU/g tissue were calculated.

### Expression of fimH, curli fiber and cellulose biosynthesis

Type 1 pili and curli fimbriae are important factors in biofilm production, so the expression of these factors was investigated in *E. coli* K46 and the four control strains. The expression of *fimH* (adhesin of type 1 pili) was tested using a yeast agglutination assay. The strains were grown in static overnight LB cultures at 37 °C. 50 μL of PBS with 1% yeast (*Saccharomyces cerevisiae*) and 150 μL of overnight culture were pipetted onto a sterile microscope slide and mixed, and the ability to agglutinate was visually observed.

The expression of curli fiber was detected by growing the strains on Congo red plates (1% tryptone agar plates containing the dye Congo red) overnight at 30 °C. Red pigment in the colonies was deemed as positive for curli fiber [14].

Since the expression of cellulose could reduce the production of biofilm [15], the bacterial strains were tested for their ability to synthesize cellulose on LB agar with 0.02  % (w/v) calcofluor white. Plates were incubated for 24 and 48 h at 30 °C, and then observed under UV light (366 nm) for the presence of fluorescence and thereby positive for the synthesis of cellulose [15].

### Growth curves and biofilm formation

The ability to produce biofilm is an advantage in environmental survival for a pathogenic strain, as well as survival within a host [16]. *E. coli* K46 and Ctr 1-Ctr 4 were tested for their ability to form biofilm in three different media; LB, M9 and YESCA. Each overnight culture was diluted 1:100 in fresh medium, and four technical replicates of 100 μL of these dilutions were transferred to a flat-bottomed polystyrene 96-well microtiter plates with lids (NUNC). The plates were incubated at 30 °C statically for 48 h, the media was withdrawn, and the wells rinsed with water to remove any planktonic bacteria. 150 μL of 0.1% crystal violet solution was added to each well and incubated for 10 min at room temperature, the solution was removed and the wells were washed with water to remove any crystal violet unbound to biofilms before the microtiter plates were air dried without a lid for 30 min. 200 μL of solvent solution (DMSO) was added to solubilize stained biofilms and the plates were covered and incubated at room temperature, while shaking for 15 min. Absorbance was obtained using a SynergyHTTR/KC4 fluorimeter/luminometer (BioTek Instruments, Inc.) at 562 nm. Three biological replicates were made for each strain. The biofilm produced was measured by arbitrary units, where the mass of biofilm produced by the various strains was compared to the mass of biofilm produced by the reference strain UTI89.

To ensure that the observations were due to the strains’ ability to form biofilm in each medium and not their ability to grow in these media, growth curves for the five strains in the three media were obtained using a Bioscreen C plate reader system (Growth Curves USA, Piscataway, NJ, USA). The setup was parallel to the biofilm protocol regarding the preparation of the cultures, but 200 μL diluted culture was used instead of 100 µL in each well. The trays were inserted into the microplate reader (Bioscreen) for 24 h with shaking, and bacterial growth was measured every 30 min at OD_600_.

### Ethics approval

Mice used in this study were handled in accordance with protocols approved by the Institutional Animal Care and Use Committee (IACUC) at the University of Utah (Protocol number 10-02014), following US federal guidelines indicated by the Office of Laboratory Animal Welfare (OLAW) and described in the Guide for the Care and Use of Laboratory Animals, 8^th^ Edition.

Zebrafish used in this study were handled in accordance with IACUC-approved protocols following standard procedures.

## Results

### *Escherichia coli* K46 is highly virulent in the zebrafish bloodstream

**Figure 1:**
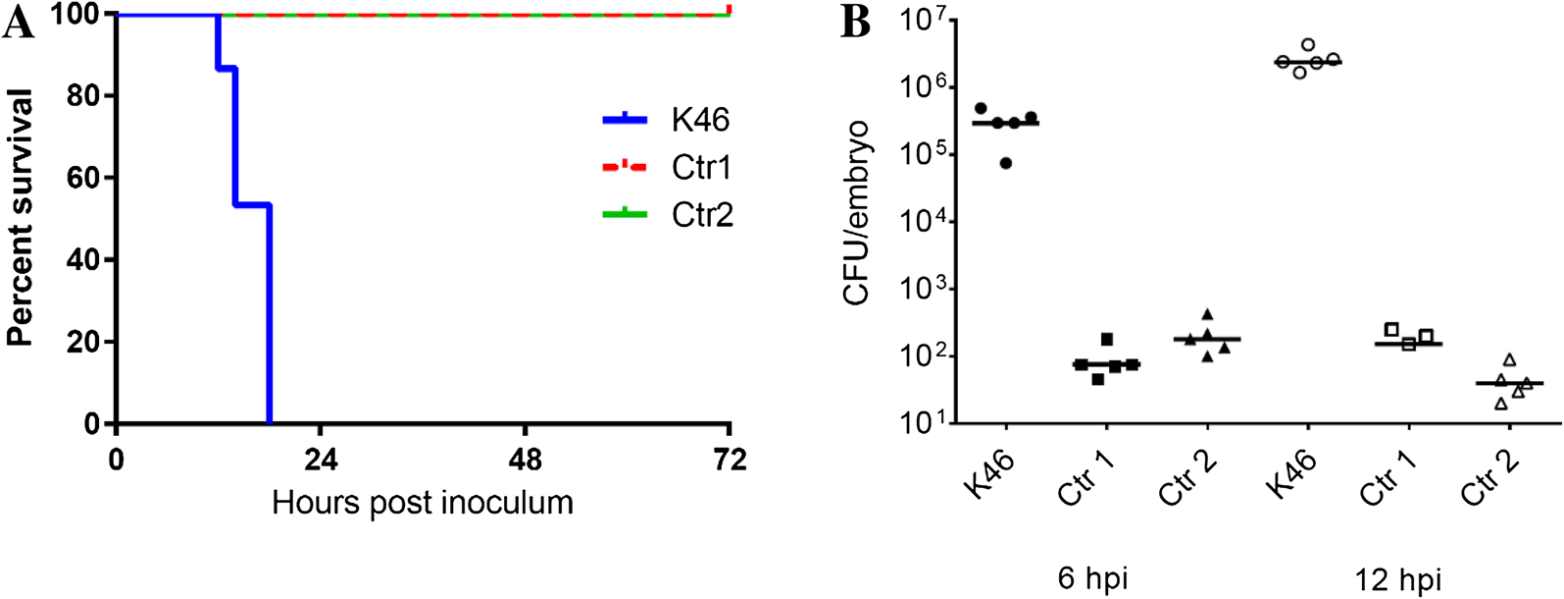
**Survival plot and corresponding bacterial titers in zebrafish embryos. A** Representative Kaplan–Meier survival plot of 48–54 h post fertilization zebrafish embryos inoculated with 10^3^ CFU of *E. coli* K46, Ctr 1 or Ctr 2. Live and dead fish were counted at 6, 12, 14, 18, 24, 48 and 72 h post inoculum. The lines for Ctr 1 and Ctr 2 coincide at 100% survival throughout the experiment. **B** The level of bacteria retrieved from the inoculated zebrafish at 6 and 12 h post inoculation (hpi). The horizontal bars indicate the median values for each group. Ctr 1 has two zero points at 12 hpi which are not graphed.

*Escherichia coli* K46 proliferated readily in the zebrafish embryos and approximately 2 and 3 magnitude higher levels of bacteria were retrieved at 6 and 12 hpi, respectively, compared to the time point of inoculation (Figure 1B). Ctr 1 and Ctr 2 did not reach higher levels in the embryos (Figure 1B), and the bacteria were eliminated within 24 hpi (data not shown). All of the embryos infected with Ctr 1 and Ctr 2 were still alive at 72 hpi (Figure 1A) and no signs of obvious disease were observed in these fish. Conversely, all the embryos challenged with *E. coli* K46 died between 12 and 18 hpi (Figure 1A).

**Figure 2:**
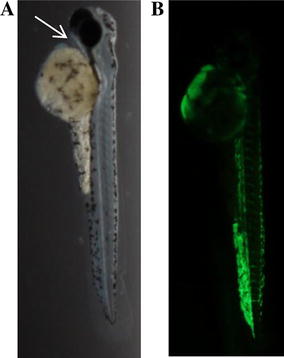
**Brightfield image of a zebrafish embryo inoculated with a fluorescent** ***E. coli*** **strain. A** Brightfield image indicating the inoculation site of *E. coli* K46/pGEN-GFP (LVA) in the circulatory valley (arrow). **B** At 12 hpi, the bacteria are located throughout the vasculature of the embryo, but with minimal intrusion into the surrounding tissues.

Infection with GFP-labeled *E. coli* K46 showed that bacteria were located throughout the vasculature at 12 hpi (Figure 2), while the zebrafish embryos inoculated with the GFP-marked Ctr 1 and Ctr 2 only showed background fluorescence at 12 hpi (data not shown). Together these results show that *E. coli* K46 is highly lethal in a zebrafish embryo model of infection and can replicate readily within the zebrafish bloodstream.

### *Escherichia coli* K46 stably colonizes the gastrointestinal tract of mice

**Figure 3:**
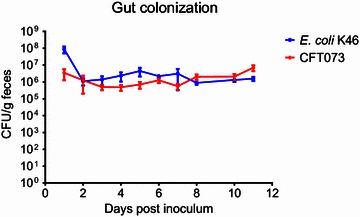
**Gastrointestinal tract colonization in adult mice.** The mice were gavaged with 1.5 × 10^8^ CFU of *E. coli* K46 (tetracycline resistant) or the reference strain UPEC strain CFT073-Clm^R^ (chloramphenicol resistant). The colonization was assessed at each of the indicated time points by enumerating CFU per gram feces of the inoculated strains. Data represent CFU/g feces ± SEM (*n* = 5 mice).

Following oral gavage into the gastrointestinal tract, *E. coli* K46 colonized the gut of all mice at an average concentration of 10^6^ CFU/g feces throughout the 12 days of experiment. This was similar to what was seen for ExPEC reference strain CFT073 (Figure 3), indicating that *E. coli* K46 is an efficient colonizer of the mouse intestine.

### *Escherichia coli* K46 colonizes the bladder poorly

**Figure 4:**
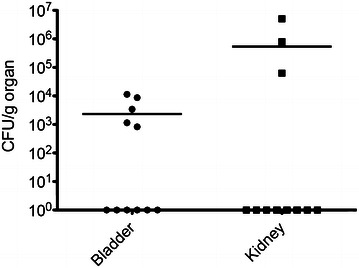
***E. coli*** **retrieved in the mural bladder and kidney following transurethral catheterization.** CFU of *E. coli* K46 present in the bladder and kidney of female CBA/J mice 3 days after transurethral catheterization of 10^5^–10^6^ CFU of *E. coli* K46. The ability to infect the organs was assessed as CFU per gram tissue. Horizontal bars denote mean values for each group (*n* = 11).

Following transurethral catheterization of K46 into the bladder, bacteria could not be detected at 3 dpi in half of the mice. In the remaining mice, 10^3^–10^4^ CFU/g tissue were isolated at this time point (Figure 4). When bacterial titers in the kidneys were examined, two of the mice with bacteria in the bladder also had 10^5^–10^7^ CFU/g bacteria present in the kidneys. A similar titer of bacteria was also found in the kidney of one mouse with no bacteria in the bladder (Figure 4). The finding of bacteria in the kidneys indicates that *E. coli* K46 has the ability to ascend in the urinary tract.

### *Escherichia coli* K46 expresses fimH, curli fiber and produces cellulose

*Escherichia coli* K46 and Ctr 1, Ctr 3 and Ctr 4 were positive for *fimH* expression as measured by yeast agglutination. *Escherichia coli* K46 and *E.* *coli* Ctr 4 were weakly positive for the expression of curli fiber and cellulose production as determined by growth on Congo Red and Calcofluor White plates (Table 2). These results suggest that *E. coli* K46 has all the requirements needed for biofilm production.

**Table 2:** **Results of fimbriae expression and cellulose synthesis**

Strains	*FimH* expression	Curli fiber	Cellulose synthesis
K46	+	+^a^	+^a^
Ctr 1	+	−	−
Ctr 2	−	−	−
Ctr 3	+	−	−
Ctr 4	+	+^a^	+^a^

The expression of *fimH* and curli fiber and the production of cellulose in *E. coli* K46 and four *E. coli* control strains Ctr 1-Ctr 4.

^a^Weak reaction.

### *Escherichia coli* K46’s ability to produce biofilm depends on the trial conditions

**Figure 5:**
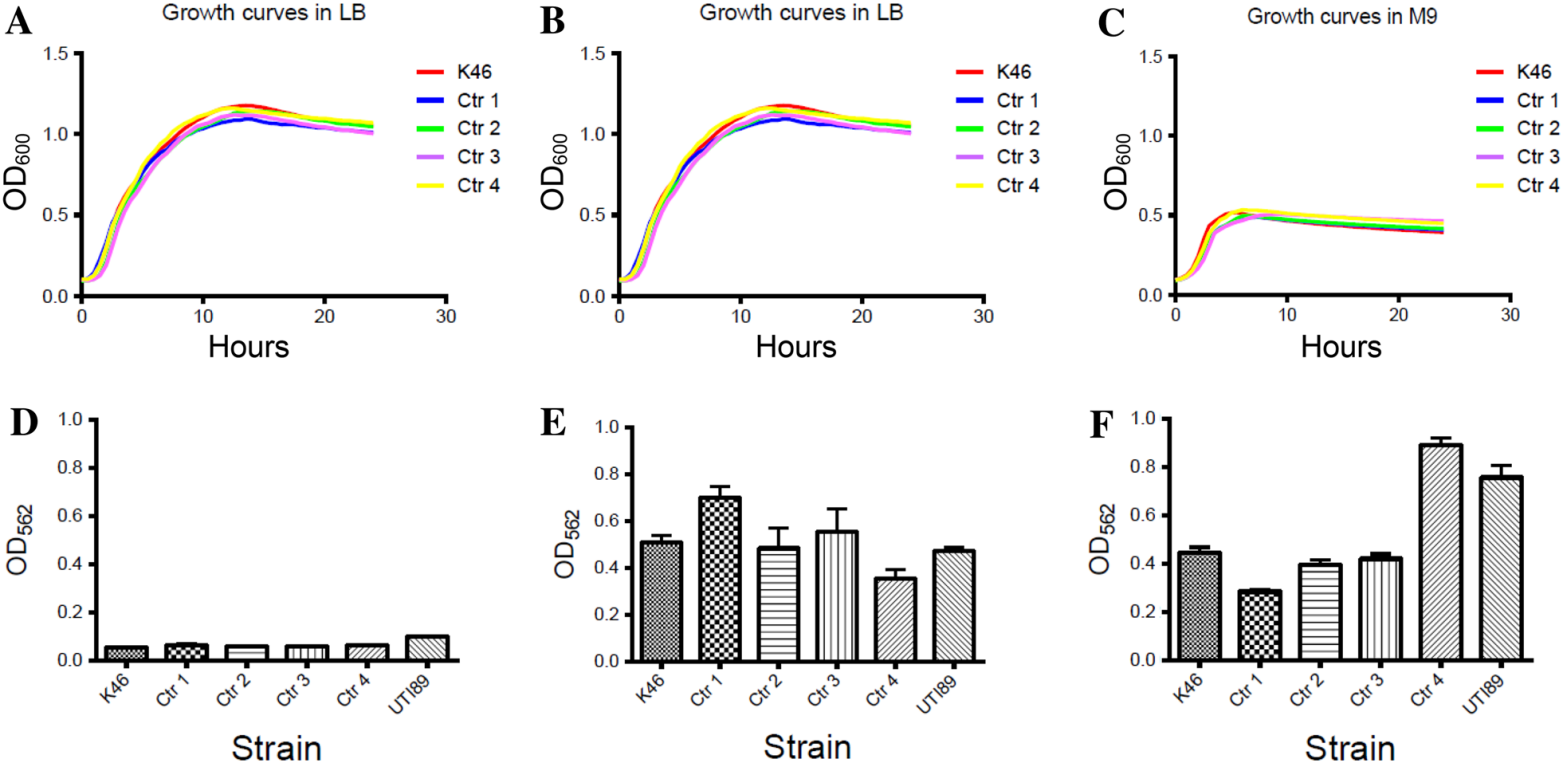
**Growth curves and biofilm formation of** ***E. coli***. **A**–**C** show representative growth curves in LB, M9, and in YESCA, respectively of *E. coli* K46 and four control strains (Ctr 1-Ctr 4). **D**–**F** show the corresponding biofilm formation, measured relative to the reference strain UTI89. All biofilm data are the mean of three independent experiments performed in quadruplicate. Error bars indicate the standard deviation.

In LB broth, both *E. coli* K46 and the control strains Ctr 1-Ctr 4 were poor biofilm producers (Figure 5A). In YESCA media, Ctr 4 formed equal amount of biofilm as ExPEC reference strain UTI89, while the other strains produced approximately 50% less (Figure 5C). In modified M9 minimal media, *E. coli* K46 and Ctr 1-Ctr 3, formed biofilm at the same or higher level than UTI89, while Ctr 4 formed approximately 20% less biofilm than UTI89 (Figure 5B). The growth characteristics of all *E. coli* strains tested were similar within each media, but differences in growth rate and final OD were observed between different media conditions (Figures 5A–C). Together this data show that *E. coli* K46 produces biofilm most efficiently under stringent conditions in vitro.

## Discussion

Fewer virulence factors (VFs) were detected in *E. coli* K46 than in half of the control strains isolated from healthy lambs, and none of the detected VFs were sufficient to explain the observed lethality in the septicemic outbreak among the neonatal lambs [12]. Despite this lack of specific VFs, we show that *E. coli* K46 is exceptionally virulent in the zebrafish model, as it killed all the infected embryos within 24 hpi, while none of the *E. coli* control strains caused any lethality. This is underscored by the zebrafish trial with the GFP marked *E. coli* K46, where the bacterial path from the infection site through the vasculature of the embryo is confirmed. The zebrafish trials correspond with the high virulence observed in the septicemic outbreak among the neonatal lambs, where all six lambs died after acute illness [12]. The clearance of the *E. coli* control strains from the zebrafish embryos is in accordance with other studies where avirulent *E. coli* K12 (DH5α) was phagocytosed and eliminated within 5 h after infection [17]. While the zebrafish results do not explain the specific mechanisms behind the microbial pathogenesis, they do support our conviction that *E. coli* K46 is highly pathogenic. Our data also support the conclusion that bacterial factors and not only host factors, contributed significantly to the lethality in the lambs.

The results obtained in the zebrafish trials further support the use of zebrafish as a suitable model organism for studying pathogenicity in *E. coli*. The zebrafish (*Danio rerio*) has a well-developed immune system, which is similar to the mammalian immune system [18]. The model is also sensitive and hence applicable for examining pathogenicity and closely related strains of *E. coli* have been shown to display differences in virulence capability in the zebrafish model which correspond with differences in pathogenicity in the human host [8].

The bacterial species in the GI tract of humans and sheep are different from those in mice, but the conditions and factors affecting bacterial competition within the mouse gut can to some extent be extrapolated to humans [19]. Nevertheless, it was somewhat surprising that *E. coli* K46 colonized the murine gut at the same high levels as the reference strain CFT073, which is recognized to be a very efficient colonizer of the mouse intestine [20]. In neonatal septicemia, the ewe may be the source of infection through contaminated fleece and udder after shedding pathogenic bacteria in the feces. The bacteria subsequently enter the lambs per os through suckling. From there the ingested bacteria likely colonize the infant gut and enter the bloodstream, resulting in septicemic disease. The portal of entry is not known in the *E. coli* K46 outbreak, but the strain’s significant ability to colonize the murine gut indicate that the gastrointestinal tract could be a possible entry route. If nothing else, the high and stable level of *E. coli* K46 in the mouse gut despite the absence of any selective pressure, shows that this strain has the essential prerequisites to outcompete an intact microbiota and colonize the gut. Whether *E. coli* K46 would establish itself as part of the normal murine gut flora permanently is not known, as the mice experiment was ended after 2 weeks duration when the level of *E. coli* K46 was still high.

Infection in the bladder and a subsequent ascending infection to the kidneys is a common infection route of septicemia in humans [21]. *E. coli* K46 colonized the murine bladder poorly, which corresponds with the lack of pathological findings in the kidneys at autopsy of the mice (data not shown). This indicates that the urogenital tract is not a preferred entry route for the entrance of *E. coli* K46 into the bloodstream, at least not in mice. In two of three mice with *E. coli* in both the kidney and the bladder, the bacteria likely reached the kidney through an ascending infection. Bacterial reflux into the kidneys during transurethral catheterization is rare [22], but this might explain the retrieval of *E. coli* only in the kidney, and not in the bladder, in one of the mice.

*Escherichia coli* K46 expressed curli fiber and the type 1 fimbriae *fimH*, in addition to possessing genes for other adhesins like *afaE VIII*, f17 fimbriae, *prfB, bmaE* and *lpfA* [12]. The expression of different fimbriae and the level of their expression may give the strain a selective advantage during colonization. Type 1 fimbriae (*fimH*) have been shown to be important in the adherence and invasion of bladder epithelial cells both in vitro and in mice [23]. Fimbriae may also play a role in the ability to form biofilm. Along with their role in the formation of secreted IgA-mediated biofilm within the gut, several groups have reported that type 1 fimbriae (*fimH*) are critical for *E. coli* biofilm formation on abiotic surfaces [24–26]. Mutations in *fimH* have been reported to reduce *E. coli* attachment to polyvinyl chloride and other abiotic surfaces [24, 27]. The expression of *fimH* has significance in the formation of biofilm in M9, while the expression of curli fiber is an important factor in biofilm formation in YESCA [28]. This is not supported by our study, where Ctr 2 (*fimH*-negative) formed biofilm at the same level as the reference strain in M9, while Ctr 4 (*fimH*-positive) did not. In YESCA, Ctr 4 formed biofilm on the same level as the reference strain, and at significant higher level than *E. coli* K46, despite both strains being weakly positive for curli fiber. In addition, a direct anti-biofilm role has been demonstrated for group II capsular polysaccharides [29], but since neither *E. coli* K46 nor Ctr 1-Ctr 4 contained genes for capsular synthesis this cannot explain the difference in biofilm production in the current study. One should keep in mind, however, that this method measures biomass and not cell viability, and that biofilm formation in vivo and in the environment are influenced by other factors [30].

Cellulose is usually produced as an extracellular component for mechanical and chemical protection and the expression of cellulose can reduce biofilm production [15]. However, the co-expression of cellulose and curli fiber can form a highly inert, hydrophobic extracellular matrix, called “bacterial wood” which surrounds the bacterial surface and function as a survival mechanism. The production of both cellulose and curli fiber in *E. coli* K46 could increase its persistence in a farm environment.

The zebrafish lethality model supports our belief that *E. coli* K46 is highly pathogenic, and that the lamb lethality seen in the septicemic outbreak was likely due to a collection of known and unknown bacterial virulence factors. The murine models show that *E. coli* K46 can stably colonize the intestinal microbiota and potentially provide a route of entry for infection. Known virulence genes essential for the high pathogenicity were not identified, but bacterial knockout mutants used in zebrafish challenge could be an interesting approach in future studies.
